# Regulatory Review Time of Vaccine Approvals in China Between 2005 and 2024

**DOI:** 10.1001/jamanetworkopen.2025.18255

**Published:** 2025-06-30

**Authors:** Yingtian Ding, Mengyuan Fu, Reshma Ramachandran, Luwen Shi, Joseph S. Ross, Xiaodong Guan

**Affiliations:** 1 Department of Pharmacy Administration and Clinical Pharmacy, School of Pharmaceutical Sciences, Peking University, Beijing, China; 2 International Research Center for Medicinal Administration, Peking University, Beijing, China; 3 Section of General Internal Medicine, Department of Internal Medicine, Yale School of Medicine, New Haven, Connecticut; 4 Collaboration for Regulatory Rigor, Integrity, and Transparency, Yale School of Medicine, New Haven, Connecticut

## Abstract

This cross-sectional study examines the regulatory review times for vaccine product applications submitted through China’s National Medical Products Administration over the past 2 decades.

## Introduction

Prompt regulatory review for vaccine marketing applications is crucial to ensuring timely public access and preventing community transmission of infectious diseases. To accelerate review time frames, China’s National Medical Products Administration (NMPA) progressively introduced regulatory pathways, including special approval, priority review, and conditional approval, to speed review of marketing authorization applications (MAAs).^1^ To better understand these processes, we characterized regulatory review times for vaccine product applications submitted to NMPA over the past 2 decades.

## Methods

This cross-sectional study was deemed exempt from review and informed consistent by the Peking University Health Science Center Institutional Review Board because no human participants or individual identifiers were involved. We followed the STROBE reporting guideline.

The study included all vaccine MAAs on the NMPA Center for Drug Evaluation (CDE) website between January 1, 2005, and December 31, 2024, identified using CDE coding principles for drug applications (eMethods in Supplement 1), including approved, declined, and still under review. The MAAs include initial vaccine, indication expansion, and change in administration route for approved vaccines. For each eligible MAA, vaccine indication, sponsor country of origin, application receipt date, approval date, and review pathway data were obtained from a commercial database and cross verified against records from CDE and NMPA.^2,3,4^

Regulatory review time was defined as the interval between NMPA application receipt and approval date. We used descriptive statistics to analyze review time by application year, review pathways, and sponsor country. Mann-Whitney *U* tests were performed to assess associations between each variable and review time. Significance was set at a 2-tailed *P* < .05. Analyses were performed using StataMP, version 18 (StataCorp LLC) and Python, version 3.11 (Vanderbilt University).

## Results

Among 272 vaccine MAAs, 201 (73.9%) were approved, 39 (14.3%) were declined, and 32 (11.8%) remained under review. Of the 201 approved MAAs, 163 (81.1%) were domestically produced and 38 (18.9%) imported; moreover, 71 (35.3%) were submitted from 2005 to 2008. The proportion approved using expedited review pathways increased from 0% from 2005 to 2008 to 56.1% from 2017 to 2020 (Figure). The most common therapeutic categories were influenza (33 [16.4%]), epidemic encephalitis (23 [11.4%]), and rabies (22 [10.9%]).

**Figure.**
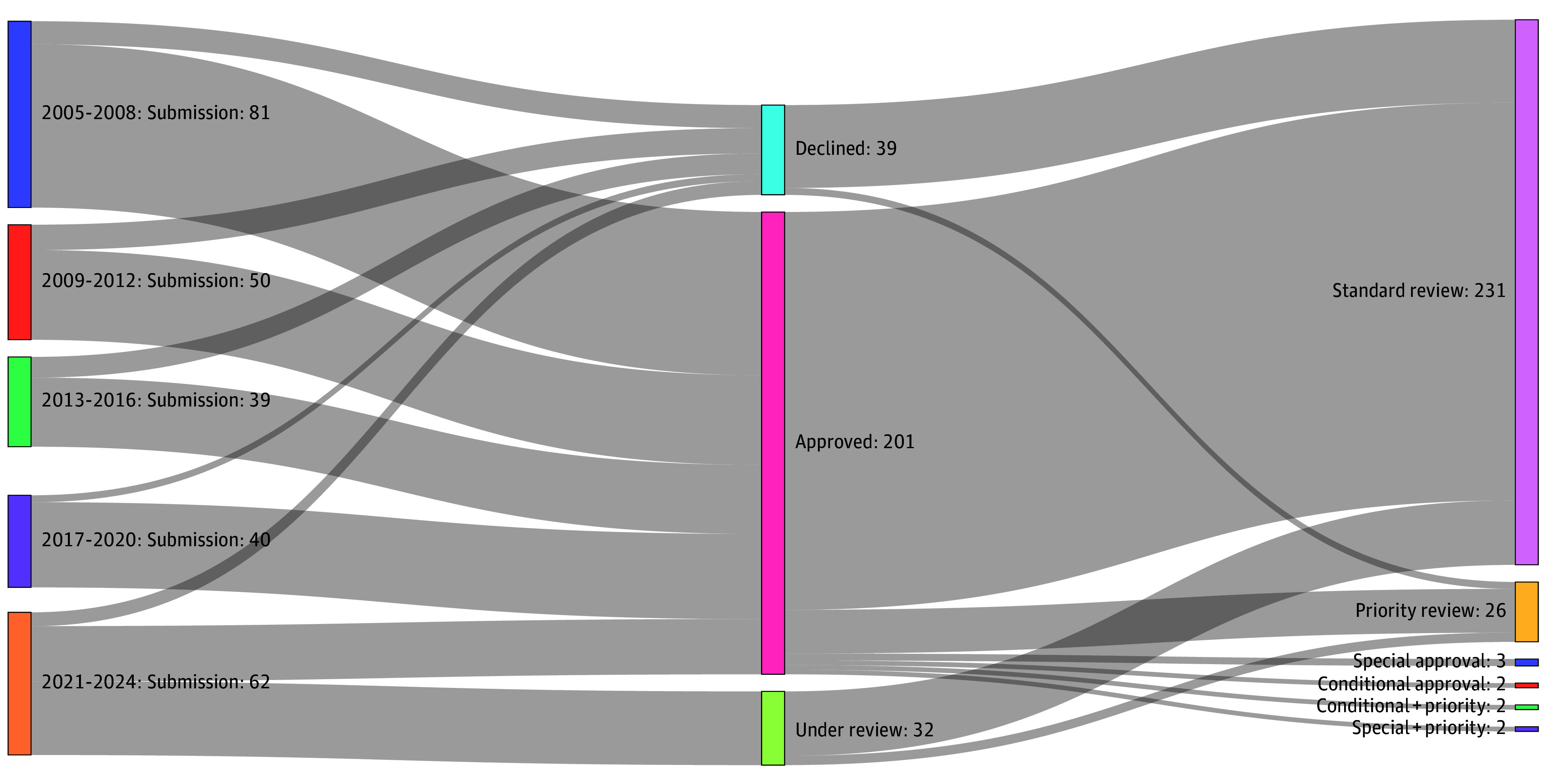
Sankey Diagram for Vaccine Applications in China, 2005-2024

The median [IQR] review time for approved vaccine MAAs was 576 (380-973) days (Table). There were no significant review time differences between domestic (median [IQR], 629 [379-1005] days) and imported (median [IQR], 503 [414-782] days) vaccines. Review times fluctuated over 4-year intervals, with the shortest time recorded between 2021 and 2024 (median [IQR], 464 [365-518] days) and longest between 2013 and 2016 (median [IQR], 978 [880-1296] days). Compared with the standard review pathway (median [IQR], 576 [411-1005] days), conditional approval was associated with significantly shorter review times (median [IQR], 8 [8-79] days; *P* = .001) but were no different for priority review (median [IQR], 629 [297-787] days) and special approval (median [IQR], 471 [161-660] days).

**Table.** Review Time Length of Approved Vaccines in China, 2005-2024

Stratified characterization	Applications, No. (%)	Length of review, median (IQR), d^a^	*P* value^b^
Total	201 (100)	576 (380-973)	NA
Manufacturer origin			
Domestic	163 (81.1)	629 (379-1005)	.35
Imported	38 (18.9)	503 (414-782)	
4-y Interval			
2005-2008	71 (35.3)	502 (355-741)	Reference
2009-2012	39 (19.4)	904 (471-1222)	.01
2013-2016	30 (14.9)	978 (880-1296)	<.001
2017-2020	37 (18.4)	502 (313-760)	.97
2021-2024	24 (11.9)	464 (365-518)	.26
Pathway^c^			
Standard review	173 (86.1)	576 (411-1005)	Reference
Priority review	23 (11.4)	629 (297-787)	.33
Special approval	5 (2.5)	471 (161-660)	.32
Conditional approval	4 (2.0)	8 (8-79)	.001
Indication			
Influenza	33 (16.4)	389 (307-471)	Reference
Epidemic encephalitis	23 (11.4)	879 (511-1191)	<.001
Rabies	22 (10.9)	577 (436-1005)	.002
Human papillomavirus	17 (8.5)	503 (439-643)	.06
Myelitis	13 (6.5)	550 (486-684)	.11
DPT	9 (4.5)	991 (441-1672)	.02
Pneumonia	9 (4.5)	782 (634-1091)	.001
H1N1 influenza	8 (4.0)	155 (111-49)	<.001
HIB	7 (3.5)	737 (498-1216)	.003
Chickenpox	7 (3.5)	702 (441-1294)	.02
Enterovirus	6 (3.0)	700 (464-910)	.01
Hepatitis B	6 (3.0)	434 (379-635)	.48
Encephalitis B	5 (2.5)	745 (507-750)	.03
Rotavirus	4 (2.0)	976 (882-1659)	.004
Tetanus	4 (2.0)	669 (278-1507)	.53
Kidney syndrome	4 (2.0)	1008 (806-1183)	.005
Hepatitis A	3 (1.5)	1098	.02
Typhoid fever	3 (1.5)	1257	.09
Herpes zoster	2 (1.0)	223	.10
Cholera	2 (1.0)	891	.06
Ebola	1 (0.5)	161	NA
Rubella	1 (0.5)	477	NA
Concretion	1 (0.5)	1133	NA
Lepra	1 (0.5)	719	NA
MMR	1 (0.5)	859	NA
Hepatitis E	1 (0.5)	660	NA
COVID-19	1 (0.5)	8	NA
Pylori	1 (0.5)	676	NA
Other combined vaccines^d^	6 (3.0)	766 (502-1236)	NA

Abbreviations: DPT, diphtheria, pertussis, and tetanus; HIB, hemophiles influenza B; MMR, measles, mumps, and rubella; NA, not applicable (data inadequate to conduct the Mann-Whitney *U* test).

^a^ For indications with insufficient data to calculate the interquartile range, only the median review days are reported.

^b^ By Mann-Whitney *U* test.

^c^ Two recombinant human papillomavirus 9-valent (types 6, 11, 16, 18, 31, 33, 45, 52, 58) vaccines were approved under both conditional approval and priority review. Mycobacterium vaccine for injection and recombinant Ebola virus disease vaccine (adenovirus type 5 vector) were approved under both special approval and priority review, counted as special approval. Those 4 vaccine applications were counted twice; therefore, the applications by pathways exceed the total.

^d^ The other 6 combined vaccines were DPT-HIB vaccines, 2 epidemic encephalitis-HIB vaccines, and 1 DPT-HIB-myelitis vaccine.

## Discussion

In this cross-sectional study of all vaccine MAAs by China’s NMPA from 2005 through 2024, median regulatory review times fluctuated but remained lengthy compared with international benchmarks, which were 6 months longer than the 12-month median review time for vaccines approved in the US between 2010 and 2020 and 2 months longer than the 16-month median for vaccines prequalified by the World Health Organization between 2009 and 2012.^5,6^ While vaccine manufacturer origin was not associated with review time, approval times using the conditional pathway were fastest. However, only 4 vaccines, for human papillomavirus, herpes zoster, and COVID-19, received conditional approval over the past 20 years in China, limiting statistical power.

This study was limited to vaccine MAAs with publicly disclosed information, which may affect generalizability. Additionally, the CDE does not distinguish initial applications, indication expansions, and administration route changes or the multiple review cycles 1 application may experience, preventing separate evaluation of these categories.

## Notes

Supplement 1. **eMethods.** Expedited Approval Pathways in China
eReferences.

Supplement 2.
Data Sharing Statement

## References

[1] Ren X, Shi L. Policy research on accelerating review and approval of new drug marketing in China, the United States and Europe. Zhongguo Xin Yao Zazhi. 2020;29(9):961-971.

[2] National Medical Products Administration of China information disclosure. Center for Drug Evaluation. Accessed January 13, 2025. https://www.cde.org.cn/main/xxgk/listpage/9f9c74c73e0f8f56a8bfbc646055026d

[3] Information inquiry. National Medical Products Administration. Accessed January 13, 2025. https://www.nmpa.gov.cn/yaopin/index.html

[4] Yaozhi Database. Accessed January 13, 2025. https://db.yaozh.com

[5] Puthumana J, Egilman AC, Zhang AD, Schwartz JL, Ross JS. Speed, evidence, and safety characteristics of vaccine approvals by the US Food and Drug Administration. JAMA Intern Med. 2021;181(4):559-560. doi:10.1001/jamainternmed.2020.7472 33170923 PMC7656319

[6] Ahonkhai V, Martins SF, Portet A, Lumpkin M, Hartman D. Speeding access to vaccines and medicines in low- and middle-income countries: a case for change and a framework for optimized product market authorization. PLoS One. 2016;11(11):e0166515. doi:10.1371/journal.pone.0166515 27851831 PMC5112794

