# Supplementary material for: Regulatory Review Time of Vaccine Approvals in China Between 2005 and 2024
Source: JAMA Netw Open. 2025 Jun 30;8(6):e2518255. doi: 10.1001/jamanetworkopen.2025.18255 (PMC12210079; doi:10.1001/jamanetworkopen.2025.18255)
Supplement: Supplement 1. — eMethods. Expedited Approval Pathways in China eReferences. [file jamanetwopen-e2518255-s001.pdf]

Supplemental Online Content

Ding Y, Fu M, Ramachandran R, Shi L, Ross JS, Guan X. Regulatory review time of vaccine approvals in China between 2005 and 2024. *JAMA Netw Open*. 2025;8(6):e2518255.
doi:10.1001/jamanetworkopen.2025.18255

eMethods. Expedited Approval Pathways in China
eReferences

This supplemental material has been provided by the authors to give readers additional information about their work.

© 2025 Ding Y et al. *JAMA Network Open*.

eMethods. Expedited Approval Pathways in China

In 2009, the National Medical Products Administration (NMPA) of China introduced special approval for new medicine with significant clinical advantages. This was followed by the implementation of priority review for medicines addressing significant clinical value, or urgent clinical and public health demands. In 2017, conditional approval was established for medicines targeting life-threatening conditions.[1] The criteria of entering the expedited pathways and its corresponding benefits in regulatory review are briefly summarized in Table 1 below.[2,3]

Table 1. Criteria and Regulatory Review Benefits of Expedited Approval Pathways in China

Priority Review	Criteria:<br>- Clinically urgent drugs in short supply, innovative drugs and improved new drugs for the prevention and treatment of major infectious diseases and rare diseases; Or<br>- New products, dosage forms and specifications of drugs for children that meet the physiological characteristics of children; Or<br>- Vaccines and innovative vaccines urgently needed for disease prevention and control; Or<br>- Drugs included in the breakthrough therapy drug program; Or<br>- Drugs that meet the conditional approval; Or<br>- Other circumstances for priority review and approval as prescribed by the State Drug Administration.<br><br>Benefit:<br>- The review time length shortened from 200 days to 130 days for priority review, and can be further shortened to 70 days if the drugs have been marketed in foreign countries but clinical urgent need for orphan disease in China<br>- Prioritize in inspecting and testing
Special Approval	Criteria:<br>- Drugs used for effectively prevent, timely control and eliminate the

© 2025 Ding Y et al. *JAMA Network Open.*

	hazards of public health emergencies, and ensure the health and life safety of the public.<br><br>Benefits:<br>- Prioritize in reviewing, inspecting and testing
Conditional Approval	Criteria:<br>- Drugs for seriously life-threatening with no effective treatment, and the clinical trial data of the drug have confirmed the efficacy with predictable clinical value;<br>- Drugs in urgent need of public health, and clinical trials of the drugs have shown efficacy with predictable clinical value;<br>- Vaccines urgently needed in response to major public health emergencies or other vaccines identified as urgently needed by the National Health Commission, whose benefits outweigh the risks after assessment.<br><br>Benefits:<br>- Products granted conditional approval may be marketed through surrogate endpoints or early clinical trial data

Study Sample and data collection

The study included all vaccine marketing applications (MAAs) disclosed on commercial database Yaozhi between January 1, 2005 and December 31, 2024. According to the CDE's coding principles for drug applications (Table 2) and ATC code, we included vaccine marketing applications acceptance number codes starting with CXSS, CYSS, JXSS, JYSS and dispensed in ATC group J07 vaccine.

Table 2. Submission Acceptance Coding Principle

Part I (4 Letters with basic information)				Part II (5th – 6th digit)	Part III (7th to 11th digit)
1st letter (Nationality)	2nd letter (Submission category)	3rd letter (Drug type)	4th letter (Submission phase)	2-digit year	5-digit serial number
**C**: Domestic	**X**: New drug	**H**: Chemical	**L**: Apply for	i.e. 05	Each category

© 2025 Ding Y et al. *JAMA Network Open*.

product<br>**J**: Import product	**Y**: Drug with national standards	medicines<br>**Z**: Chinese medicines or natural medicines<br>**S**: Biologics<br>**F**: Excipients	clinical trial<br>**S**: Apply for marketing registration<br>**B**: Supplement<br>**Z**: Renew<br>**F**: Sub-package	represents the year of 2005	starting from 00001

By searching Yaozhi database with the specified search criteria, 279 applications were initial identified. Applications that were not for vaccines, or that lacked an acceptance date or review status date, were excluded from the study (Figure 1). While analyzing the review time length, 201 MAAs were included after excluding the applications still under review and the applications being declined as rejection or withdrawn may happen at any time during the review. All data were cross verified using CDE and NMPA website.

Figure 1. Flowchart of Vaccine Marketing Application Inclusion and Exclusion

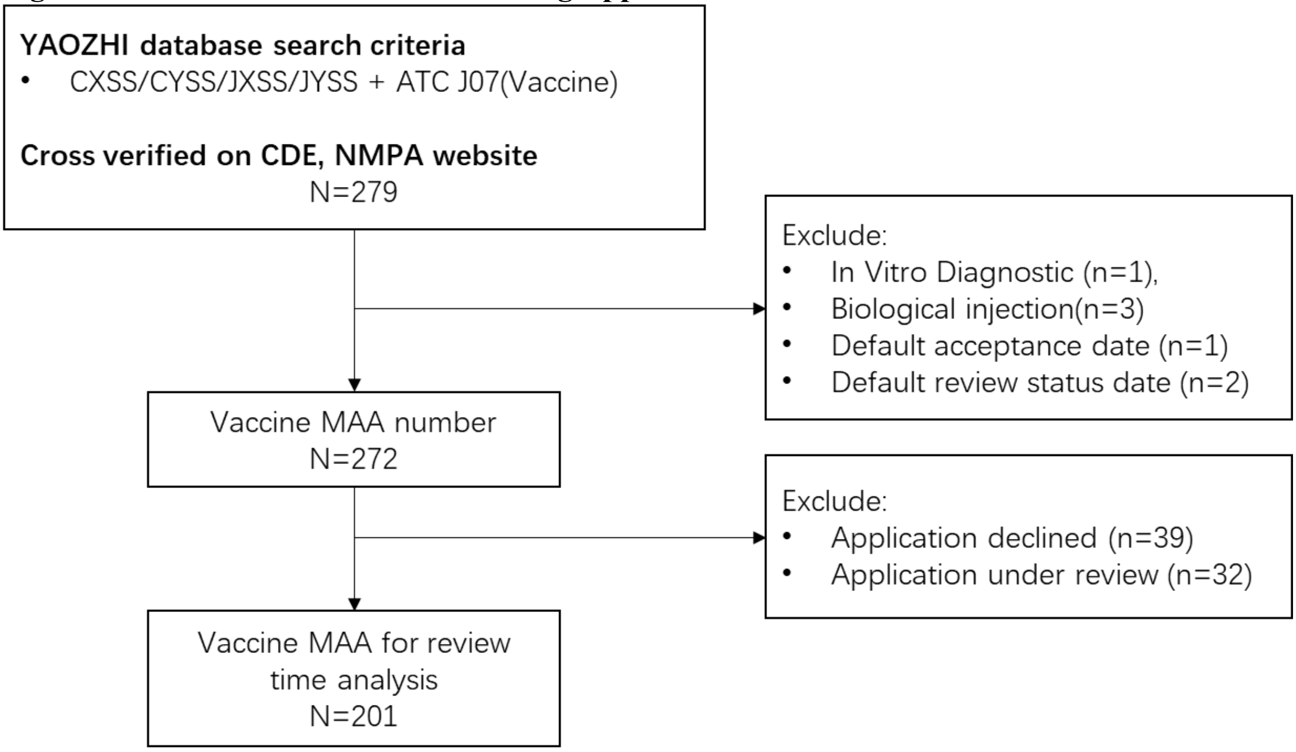

© 2025 Ding Y et al. *JAMA Network Open*.

eReferences

1. Ren X, Shi L. Policy research on accelerating review and approval of new drug marketing in China, the United States and Europe. Chinese Journal of New Drugs. 2020;29(9):961-971
2. National Medical Products Administration, Announcement of the State Food and Drug Administration on the release of three documents including the "Breakthrough Therapy Drug Review Procedures" (No. 82 of 2020). https://www.nmpa.gov.cn/xxgk/fgwj/xzhgfxwj/20200708151701834.html. Accessed April 15, 2025
3. National Medical Products Administration, State Food and Drug Administration drug special approval procedures. https://www.samr.gov.cn/cms_files/filemanager/samr/www/samrnew/samrgkml/nsjg/bgt/202106/W020211125329887725524.pdf. Accessed April 15. 2025

© 2025 Ding Y et al. *JAMA Network Open.*
