# Supplementary material for: Regulatory Review Time of Vaccine Approvals in China Between 2005 and 2024
Source: JAMA Netw Open. 2025 Jun 30;8(6):e2518255. doi: 10.1001/jamanetworkopen.2025.18255 (PMC12210079; doi:10.1001/jamanetworkopen.2025.18255)
Supplement: Supplement 2. — Data Sharing Statement [file jamanetwopen-e2518255-s002.pdf]

# Data Sharing Statement

Ding. Regulatory Review Time of Vaccine Approvals in China Between 2005 and 2024. *JAMA Netw Open*. Published June 30, 2025. doi:10.1001/jamanetworkopen.2025.18255

**Data**
**Data available:** No

**Additional Information**
**Explanation for why data not available:** Relevant data are available on reasonable request from the corresponding author.
